# Genomic comparisons of *Streptococcus suis* serotype 9 strains recovered from diseased pigs in Spain and Canada

**DOI:** 10.1186/s13567-017-0498-2

**Published:** 2018-01-09

**Authors:** Han Zheng, Pengchen Du, Xiaotong Qiu, Anusak Kerdsin, David Roy, Xuemei Bai, Jianguo Xu, Ana I. Vela, Marcelo Gottschalk

**Affiliations:** 1 State Key Laboratory of Infectious Disease Prevention and Control, Collaborative Innovation Center for Diagnosis and Treatment of Infectious Diseases, National Institute for Communicable Disease Control and Prevention, Chinese Center for Disease Control and Prevention, Changping, Beijing, China; 2 Beijing Key Laboratory of Emerging Infectious Diseases, Institute of Infectious Diseases, Beijing Ditan Hospital, Capital Medical University, Beijing, China; 3 Faculty of Public Health, Kasetsart University Chalermphrakiat Sakon Nakhon Province Campus, Bangkok, Sakon Nakhon Thailand; 4 Swine and Poultry Infectious Diseases Research Center, Faculty of Veterinary Medicine, University of Montreal, Montreal, QC Canada; 5 Departamento de Sanidad Animal, Facultad de Veterinaria and Centro de Vigilancia Sanitaria Veterinaria (VISAVET), Universidad Complutense de Madrid, Madrid, Spain

## Abstract

*Streptococcus suis* is one of the most important bacterial pathogens in the porcine industry and also a zoonotic agent. Serotype 9 is becoming one of the most prevalent serotypes within the *S. suis* population in certain European countries. In the present study, serotype 9 strains isolated from a country where infection due to this serotype is endemic (Spain), were compared to those recovered from Canada, where this serotype is rarely isolated from diseased pigs. For comparison purposes, strains from Brazil and the only strain isolated from a human case, in Thailand, were also incorporated. Firstly, sequence types (STs) were obtained followed by detection of putative virulence factors. Phylogenetic trees were constructed using the non-recombinant single nucleotide polymorphisms from core genomes of tested strains. Most Spanish strains were either ST123 or ST125, whereas Canadian strains were highly heterogeneous. However, the distribution of putative virulence factors was similar in both groups of strains. The fact that ST16 strains harbored more putative virulence genes and shared greater similarity with the genome of human serotype 2 strains suggests that they present a higher zoonotic and virulence potential than those from Canada and Spain. More than 80% of the strains included in this study carried genes associated with resistance to tetracycline, lincosamides and macrolides. Serotype 9 strains may be nearly 400 years old and have evolved in parallel into 2 lineages. The rapid population expansion of dominant lineage 1 occurred within the last 40 years probably due to the rapid development of the porcine industry.

**Electronic supplementary material:**

The online version of this article (10.1186/s13567-017-0498-2) contains supplementary material, which is available to authorized users.

## Introduction

*Streptococcus suis* is one of the most important pathogens in the porcine industry causing septicemia with sudden death, meningitis, and a variety of other diseases [1]. *S. suis* is also an important zoonotic agent afflicting people in close contact with infected pigs or pork-derived products. Thirty-five serotypes (types 1-34 and 1/2) have been described based on capsular polysaccharides [2]. Recent studies have suggested that serotypes 20, 22, 26, 32, 33, and 34 do not belong to the *S. suis* species [3, 4]. However, more serotypes remain to be described [5]. Serotype 2 is by far the most common serotype isolated from swine and human cases [1, 6]. However, serotype 9 has become an important and prevalent serotype causing invasive disease in pigs in many European countries [6, 7]. This serotype has been frequently isolated from diseased animals in intensively-reared commercial pig breeds, particularly in Spain, Germany, and the Netherlands [6]. Interestingly, it has also been isolated from Iberian pigs reared outdoors, wild boars, and even wild rabbits in Spain [8–10]. Epidemiological studies suggest that the prevalence of serotype 9 has also increased in China over recent years [11] and, to a much lesser extent, in North America (Canada) [12]. Until recently, no potential zoonotic properties had been attributed to this serotype. However, a human case due to *S. suis* serotype 9 was recently reported in Thailand [13].

The new genomic techniques have enabled a qualitative leap in the research of virulence factors as well as the pathogenesis of the infection caused by *S. suis* [14–16]. Significant genomic differences within the *S. suis* population, even amongst strains belonging to the same serotype, implied that virulence potential is genetically related. For example, it is well known that serotype 2 strains from Europe and Asia are genotypically and phenotypically different from those of North America [17] and have a different virulence potential [18]. In addition, different virulence factors have been proposed for this serotype and some of these are exclusively present among strains of a given geographical area [19]. On the other hand, few data are available for serotype 9 strains, despite the growing importance of this serotype as a swine pathogen; in fact, only a few studies have reported that not all serotype 9 strains have the same virulence potential [20–22], although it is generally accepted that strains of this serotype are less virulent than those of serotype 2 after experimental intranasal infections [21, 23].

Since it has been clearly shown that serotype 2 strains from Europe and North America are highly different [18, 24], it was of interest to evaluate if such observation may also be applied to *S. suis* serotype 9. To answer this question, strains isolated from diseased pigs in Spain and Canada, where serotype 9 is high or low prevalent, respectively, were analyzed in the present study. For comparison purposes, available strains from Brazil as well as the sole strain isolated from a human patient (Thailand) [13] were also included. Sequence types (STs) were obtained after multilocus sequence typing (MLST) analysis and the presence of previously described putative virulence factors determined. Comparative genomic analysis (CGA) of selected strains was further carried out and phylogenic studies were done by constructing a phylogenetic tree using the non-recombinant single nucleotide polymorphisms (SNPs) from core genomes of tested strains.

## Materials and methods

### Bacterial strains and chromosomal DNA preparation

A total of 66 *S. suis* serotype 9 strains isolated from pigs with invasive disease were included in this study. Strains from a given country originated from non-related farms. Strains were isolated in pure culture or as predominant pathogen and considered to be involved in pathological processes. Of them, 40 strains were from Spain and 23 from Canada. For comparison purposes, the sole serotype 9 strain isolated from a human patient so far—strain 1584695, a case of septic shock in Thailand—as well as 3 strains from Brazil were also included. The list of strains is presented in Table 1. The serotype of tested strains was determined by co-agglutination [25] and confirmed by a capsular gene PCR typing system [26]. Chromosomal DNA was prepared from all isolates using the method previously described [27]. The finished genome sequences of the highly virulent strain GZ1 (CP000837, serotype 2, China) [28], strain D12 (CP002644, serotype 9, China) [16], strain DN13 (CP015557, serotype 9, China), and assembled genome sequences of the strain S91K (ERS132522, serotype 9, UK) [14], the serotype 9 reference strain 22083 (PRJNA171418, Denmark) [15], and the intermediate virulent strain 89-1591 (PRJNA171430, serotype 2, Canada) [15, 28], available in public databases, were used in this study for comparison purposes.

**Table 1:** **Information of the 30 sequenced** ***S. suis*** **serotype 9 strains included in this study**

Country	Strains ID	MCG	ST	Source	Organ	Year of isolation	Genome accession no.	*mrp*	*epf*	*sly*	*cps* type
Spain	40	3	ST125	Diseased pig (meningitis)	Brain	1999	SRS1750970	NA1	−	+	1
	C04/1236-03L1	3	ST123	Diseased pig (meningitis	Brain	2004	SRS1750971	NA1	−	+	1
	746/02	3	ST125	Diseased pig (meningitis)	Brain	2002	SRS1750972	NA1	−	+	1
	BA05/00440-03	3	ST123	Diseased pig (meningitis)	Brain	2005	SRS1750973	NA1	−	+	1
	BA05/00094-01Z1	3	ST123	Diseased pig (meningitis)	Brain	2005	SRS1750974	NA1	−	+	1
	C04/1208/01C1	3	ST123	Diseased pig (meningitis)	Brain	2004	SRS1750975	NA1	−	+	1
	C04/1428-04P1	3	ST791	Diseased pig (pneumonia)	Lung	2004	SRS1750976	NA1	−	+	1
Brazil	1135/10	7-3	ST730	Diseased pig (septicemia)	Spleen	2010	SRS1750977	–	−	−	1
	1136/10	7-3	ST730	Diseased pig (meningitis)	Brain	2010	SRS1750978	–	−	−	1
	1016/10	1	ST16	Diseased pig (meningitis)	Brain	2010	SRS1750979	NA2	−	+	1
Thailand	1584695	1	ST16	Patient (septicemia)	Blood	2013	SRS1751398	NA2	−	+	1
Canada	89-289	7-3	ST731	Diseased pig (pneumonia)	Lung	1989	SRS1750983	–	−	−	2
	1509635	N	ST732	Diseased pig (pneumonia)	Lung	2013	SRS1751384	–	−	−	1
	1439272	7-1	ST788	Diseased pig (pneumonia)	Lung	2012	SRS1751385	NA3	−	−	1
	1398038	7-1	ST788	Diseased pig (septicemia)	Spleen	2012	SRS1751386	NA3	−	−	1
	1358915	4	ST733	Diseased pig (septicemia)	Spleen	2012	SRS1751387	EU	−	−	1
	1275845	7-2	ST734	Diseased pig (meningitis)	Brain	2011	SRS1751389	–	−	−	2
	1273590	7-3	ST622	Diseased pig (pneumonia)	Lung	2011	SRS1751390	–	−	+	1
	1135776	7-1	ST788	Diseased pig (pneumonia)	Lung	2009	SRS1751391	NA3	−	−	1
	1137833	7-3	ST621	Diseased pig (meningitis)	Brain	2009	SRS1751392	–	−	+	1
	1130349	7-1	ST788	Diseased pig (meningitis)	Brain	2009	SRS1751393	NA3	−	−	1
	1129705	7-1	ST788	Diseased pig (meningitis)	Brain	2008	SRS1751394	NA3	−	−	1
	1092236	N	ST54	Diseased pig (meningitis)	Brain	2008	SRS1751396	EU	−	+	1
	1388970	7-3	ST623	Diseased pig (pneumonia)	Lung	2012	SRS1751399	–	−	−	2
	1679718	7-3	ST621	Diseased pig (pneumonia)	Lung	2014	SRS1751400	–	−	+	1
	74911-8	7-2	ST735	Diseased pig (septicemia)	liver	2015	SRS1751402	–	−	−	1
	1778687	7-3	ST789	Diseased pig (septicemia)	Spleen	2015	SRS1751403	–	−	−	1
	1803662	7-3	ST622	Diseased pig (septicemia)	liver	2015	SRS1751404	–	−	+	1
	1808171	7-3	ST220	Diseased pig (septicemia)	Spleen	2015	SRS1751414	–	−	−	2
	1814305	7-3	ST790	Diseased pig (meningitis)	Brain	2015	SRS1751415	–	−	−	1

### Multilocus sequence typing (MLST) and minimum core genome (MCG) typing

MLST and MCG were performed using PCR amplification and DNA sequencing as previously described [29, 30].

### Sequencing and bioinformatics analysis

Based on MLST results, geography and isolation time, 19 Canadian strains, 7 Spanish strains, the 3 Brazilian strains, as well as the human strain from Thailand (a total of 30 strains) were selected for Illumina sequencing and sequences were assembled into contigs and scaffolds using SOAPdenovo (release1.04) [15]. Genes were predicted using Glimmer and gene orthologs were determined using OrthoMCL [15].

### Comparative genomic analysis

Comparative genomic analysis (CGA) of the 30 selected strains from this study were first carried out using the highly virulent serotype 2 strain GZ1 [28] as reference strain. Sequences were also compared to the serotype 2 intermediately virulent Canadian strain 89-1591 [28], as well as the serotype 9 strains D12, DN13, S91K, and 22083. Genes having a global match region at <80% of the nucleotide acid sequence with an identity of <80% were determined as absent compared to the genes in the highly virulent serotype 2 GZ1 strain [14]; otherwise, the gene was deemed present. The presence or absence of a gene was coded as binary data with gene presence as 1 and absence as 0. The phylogenetic tree using average linkages (UPGMA) was calculated and constructed with R (version 2.15.3) and Qiim (version 1.7). Tree was presented using FigTree (version 1.4.3). Each *cps* locus sequence was compared to that of *S. suis* serotype 9 reference strain 22083 (Genbank accession No. BR001006) [31]. The genes having a global match region at >50% of the amino acid sequence and with an identity of >50% were identified to be same homology groups (HG) [31]. The Artemis comparison tool was used to visualize the compared data [32]. Antimicrobial gene-resistance analysis was carried out by searching the antibiotic resistance genes database [33]. A resistance gene was only regarded as a homolog in tested strains if it showed at least 80% identity in protein sequence across 80% of the length of the protein [34].

### Analysis of phylogeny

The phylogeny of the 30 sequenced strains from this study as well as four additionally available serotype 9 genomes (D12, DN13, S91K, and 22083) was assessed. Genes that were included in all strains were considered to be the core genome. Mobile genes were excluded from the core genome according to the method described in a previously study [35]. Single-nucleotide polymorphisms (SNPs) in core genome were detected using SOAPsnp v1.03 and MUMmer v3.23. Gene segments with recombination in the 34 genomes were identified using a method previously described [15, 36]. The SNPs in relevant portions of the recombined regions were removed. The mutational SNP sites were then selected to construct phylogenetic trees using the Bayesian evolutionary method by BEAST v1.8.2. The best-fit model for the dataset was the TN93 substitution model, with a lognormal distribution of the substitution rate. We performed the analysis with sampling every 10 000 generations of 100 000 000 Markov chain Monte Carlo chains. Tree was presented using FigTree (version 1.4.3).

### Nucleotide sequence accession number

Sequence of *mrp*^*NA3*^, newly observed in this study, was deposited in Genbank under the accession number KY689073. Sequences of *cps* loci were deposited in Genbank under the accession number KY574604 (strain C04/1428-04P1, type 1) and KY574603 (strain 1388970, type 2). Sequences of ICEs and genomic islands obtained in this study were deposited in Genbank under the accession number KY400494 (ICESsu_1439272_), KY400495 (ICESsu_1679718_), and KY400496 (ΦSsu_1135/10_). Reads of newly sequenced strains obtained in this study were deposited in Genbank under the 30 corresponding accession numbers that can be found in Table 1.

## Results

### MLST

First, 67 serotype 9 strains isolated from 4 countries (Spain, Canada, Brazil, and Thailand) were analyzed by MLST (Table 1). High homogeneity was found within the 40 Spanish strains, of which the ST123 (*n* = 18) and ST125 (*n* = 21) predominated, with only one strain being ST791. It is noteworthy that only the *gki* sequence was different among the three STs which are all derived from the clonal complex (CC) 61 [37]. Compared to the Spanish strains, a considerably high heterogeneity was found within the 23 Canadian strains. Thirteen STs were identified, with ST788 being the predominate ST (*n* = 8). With the exception of ST621 (*n* = 3) and ST622 (*n* = 2), ST54, ST220, ST623, ST731, ST732, ST733, ST734, ST735, ST789, and ST790 contained only a single strain each. One Brazilian strain (1135/10) was ST16 (belonging to the CC16), identical to that of the Thai strain 1584695 recovered from a human patient. The other two Brazilian strains were ST730. As described in Materials and methods, based on MLST results, geographic origin and isolation time, 30 strains were further selected for Illumina sequencing.

### Difference in virulence potential among tested strains

**Figure 1:**
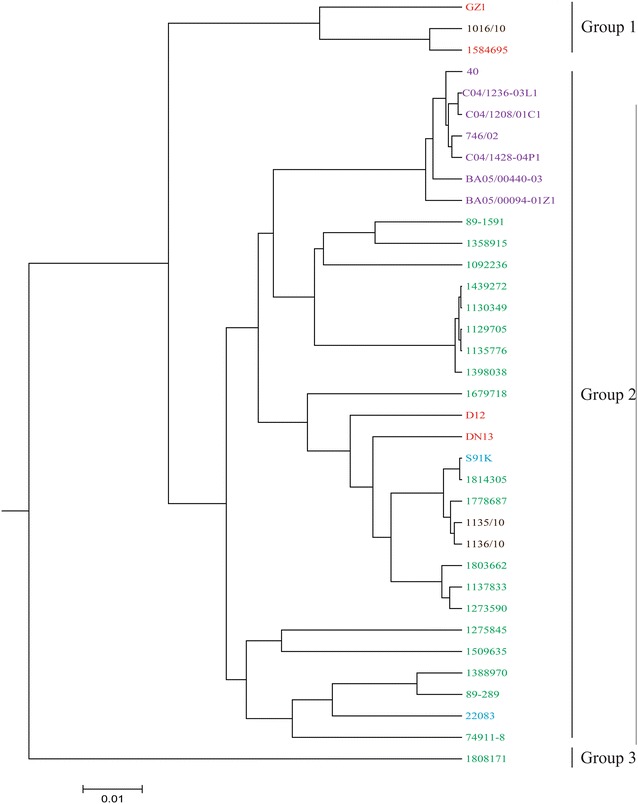
**Phylogenetic relationship of the 34** ***S. suis*** **serotype 9 strains, the intermediately virulent serotype 2 strain 89-1591, and the highly virulent serotype 2 reference strain GZ1 following CGA.** Absence or presence of each gene from each strain was translated to 0 or 1, respectively. Colors represent geographical origin of strains: Red: Asia; Brown: Brazil; Green: Canada; Purple: Spain; Blue: European countries (other than Spain).

We then evaluated the virulence potential of the 30 *S. suis* serotype 9 strains by comparing their genomes to that of the highly virulent serotype 2 ST1 GZ1 and intermediate virulent ST25 89-1591 strains by CGA. For comparison purposes, the genomes of additionally published serotype 9 strains (S91K, D12, DN13, and 22083) were also included. CGA-based phylogeny distributed all of the strains into three groups (Figure 1). Group 1 contained the Brazilian ST16 and Thai strains, along with the highly virulent ST1 strain GZ1. These strains seem to have a certain zoonotic potential, which was confirmed by the fact that the Thai strain was indeed isolated from a human case. However, within the group, the two serotype 9 strains were present in a separate branch when compared to the serotype 2 strain GZ1 (Figure 1).

Group 2 consisted of 27 strains, including all 7 Spanish strains, the two remaining Brazilian strains, and 18 Canadian strains, which were included in the same group as the four published serotype 9 genomes. They all clustered with the intermediate virulent serotype 2 ST25 strain 89-1591, distant from strain GZ1. The Canadian serotype 9 strain 1808171 clustered into group 3 and was distinct from the other 2 groups.

### Distribution of putative virulence-related genes among the *S. suis* serotype 9 strains

To further correlate the potential virulence of strains with specific genes, the presence of 57 putative virulence genes (excluding *mrp*, *sly*, and *epf*) as previously reviewed [17, 38] were studied in the 30 serotype 9 genomes (including the four published genomes) used in CGA. Indeed, 28 of them were present in all 34 tested serotype 9 genomes (Additional file 1). However, *nadR* (SSGZ1_1789) and *ofs* (SSGZ1_1494), present in highly pathogenic serotype 2 strains (including GZ1) [39], were absent in all serotype 9 strains tested (Additional file 1). Virulence genes which were preferentially present in Brazilian ST16 strain 1016/10 and Thai strain 1584695 were the *virB4* (SSGZ1_0481), *virD4* (SSGZ1_0487), *sao* (SSGZ1_1217), and *revS* (SSGZ1_1897) genes (Additional file 1).

Additionally, the regions of difference (RD), which were previously identified to be preferentially present in highly pathogenic strains [39] were also investigated in the 34 serotype 9 genomes (excluding RD17 (SSGZ1_0555–SSGZ1_0577 coding serotype 2 *cps* locus, normally absent in serotype 9 strains). Amongst these, RD6 (SSGZ1_0164–SSGZ1_0166), RD12 (SSGZ1_0342–SSGZ1_0346), RD29 (SSGZ1_0848–SSGZ1_0849), RD40 (SSGZ1_1285–SSGZ1_1288), and RD53 (SSGZ1_1787–SSGZ1_1791) were absent from all 34 tested serotype 9 strains. Only RD14 (SSGZ1_0410–SSGZ1_0414), RD21 (SSGZ1_0682–SSGZ1_0690), and RD60 (SSGZ1_1903–SSGZ1_1911) were present in both the Brazilian ST16 strain 1016/10 and Thai strain 1584695 (Additional file 2), which were closer to GZ1 in CGA.

In addition, the presence of *mrp*, *epf*, and *sly* genes, considered “classical” virulence markers mostly described for serotype 2 strains [40], were studied. Interestingly, none of the serotype 9 strains was *epf*^+^ (Table 1). However, all Spanish strains, the Brazilian ST16, and the human Thai strains were *sly*^+^. Conversely, only 3 of the 23 Canadian strains were positive for the latter gene (Table 1). Seven Spanish, 7 Canadian, 1 Brazilian, and 1 Thai strains contained putative full-length *mrp* gene copies. Interestingly, all Spanish strains studied carried the *mrp*^NA1^ genotype, while the Brazilian ST16 strain 1016/10 and Thai strain 1584695 carried the NA2 genotype [41]. EU genotype was also found in 2 Canadian strains. In the present study, we also found a new genotype of *mrp* in 5 Canadian strains, named *mrp*^NA3^. Compared to the NA1, NA2, and EU genotypes, variations were mainly present in the 5′ side region of the NA3 genotype (from the 221^st^ to the 580^th^ amino acids).

There were seven genotypes of *mrp*, *epf*, and *sly* amongst the serotype 9 strains: *mrp*^NA1^ *epf*^−^*sly*^+^ (*n* = 40); *mrp*^−^ *epf*^−^*sly*^−^ (*n* = 13); *mrp*^NA3^ *epf*^−^*sly*^−^ (*n* = 5); *mrp*^−^ *epf*^−^*sly*^+^ (*n* = 5); *mrp*^NA2^ *epf*^−^*sly*^−^ (*n* = 2); *mrp*^EU^ *epf*^−^*sly*^−^ (*n* = 1), and *mrp*^EU^ *epf*^−^*sly*^+^ (*n* = 1). All strains from Spain were genotyped as *mrp*^NA1^ *epf*^−^*sly*^+^. The Brazilian and Thai ST16 strains 1016/10 and 1584695 were genotyped as *mrp*^NA2^ *epf*^−^*sly*^+^. Five other genotypes were also found in Canadian strains.

### Detection of drug resistance genes

In order to investigate the presence of antibiotic resistance genes, sequences of the 30 strains were analyzed using the antibiotic resistance genes database. Genes coding for resistance to tetracyclines, macrolides, aminoglycosides and/or lincosamide were detected. Eleven of 19 tested Canadian strains carried at least three antibiotic resistance genes, a proportion higher than that of the 7 tested strains from Spain, of which only one strain (C04/1428-4P1) carried four antibiotic resistance genes (Table 2).

**Table 2:** **Drug resistance genes identified in 30** ***S. suis*** **serotype 9 sequenced strains**

Antibiotic resistance genes	No of strains	Geographical origin (number of strains)
None	2	Spain (*n* = 1), Canada (*n* = 1)
*tet*(W)	1	Thailand
*tet*(W), *lnub*	1	Brazil
*tet*(M), *lnub*	1	Canada
*tet*(O), *ermb*	11	Spain (5), Canada (6)
*ermb*, *lnub*, *ant6ia*	1	Canada
*tet*(O), *ermb*, *lnub*	6	Canada
*tet*(O), *ermb*, *ant6ia*	1	Canada
*tet*(O), *ermb*, *lnub*, *ant6ia*	1	Canada
*tet*(O), *tet*(*L*), *ermb*, *aph3*-*iiia*, *lnub*	1	Canada
*tet*(O), *ermb*, *aph3*-*iiia*, *ant6ia*	2	Spain (1), Brazil (1)
*tet*(O), *ermb*, *lnub*, *ant6ia*	1	Canada
*tet*(O), *ermb*, *aph3*-*iiia*, *ant6ia*	1	Brazil

### Genes coding for resistance to tetracyclines

**Figure 2:**
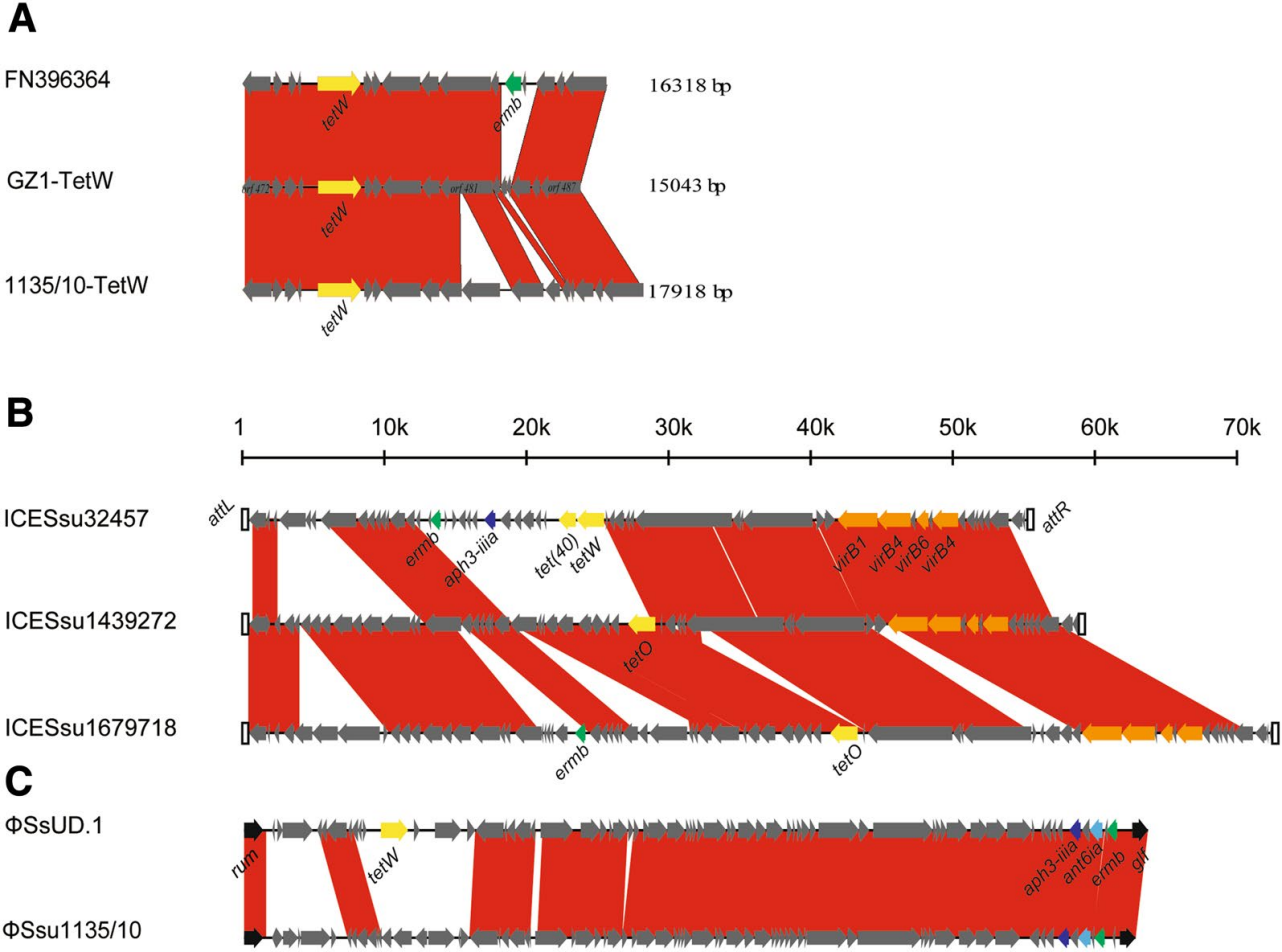
**Comparison of the gene islands or ICEs reported in this study and those found in previous studies. B** and **C** used common ruler of size. Each arrow represents a gene. Drug resistance genes were indicated in different colors. **A** Comparisons of molecular characterization of the *tet(W)*-carrying genetic element. **B** Comparisons of ICEs. Integration and conjugation genes were also indicated in different colors. **C** Comparisons of phages. Two phages were inserted in 3 side of *rum.*

Four types of genes coding for resistance to tetracyclines were found in 27 strains, including *tet*(M), *tet*(O), *tet*(L), and *tet*(W). Only 3 strains did not carry any tetracycline resistance genes, one of them from Spain and other two from Canada. One Canadian strain (74911-8) carried two tetracycline resistance genes: *tet*(O) and *tet*(L). *tet*(O) was the prevalent tetracycline resistance determinant in this study, being present in 24 strains. *tet*(M), *tet*(W), and *tet*(L) were also found in one, two, and one strain, respectively. It is noteworthy that the tetracycline resistance determinant found in the ST16 strains 1016/10 and 1584695 was *tet*(W), identical to that of strain GZ1. The molecular characterization of the *tet*(W)-carrying genetic element in these strains were further investigated. Both of these were located in nontransferable genomic islands highly similar to that of the serotype 2 strain GZ1 (GI_GZ1_, SSGZ1_465 to SSGZ1_508, ~47 kb). In fact, they only differed from GI_GZ1_ in the absence of two genes (SSGZ1_483 and SSGZ1_506) and insertion of *orf*11 (putative reverse transcriptases) and *orf*13 (hypothetical protein) (Figure 2A).

### Genes coding for resistance to macrolides, streptogramin, aminoglycosides and lincosamides

Macrolides, streptogramin_b and lincosamides resistance gene *ermb* (rRNA adenine *N*-6-methyltransferase), streptomycin resistance gene *ant6ia* (aminoglycoside *O*-nucleotidyltransferase), aminoglycosides resistance gene *aph3*-*iiia* (aminoglycoside *O*-phosphotransferase) and lincosamides resistance gene *lnub* (lincosamide nucleotidyltransferase) were found in 25, 7, 4 and, 12 strains, respectively.

### Integrative and conjugative elements (ICEs)

In this study, we found 2 novel ICEs and 1 phage, named ICESsu_1439272_, ICESsu_1679718_, and ΦSsu_1135/10_. ICESsu_1439272_ was present in five Canadian strains: 1439272, 1398038, 1135776, 1130349, and 1129705. ICESsu1679718 was found in two Canadian strains: 1679718 and 1137833.

ICESsu_1439272_ and ICESsu_1679718_ were integrated into the genome between the 50S ribosomal protein L7/L12 (*rplL*) and the *rum* genes. ICESsu_1439272_ carried the *tet*(O) gene whereas ICESsu_1679718_ carried the *tet*(O) and *ermb* genes. They only differed from the previously described ICESsu_32457_ of a non-typable *S. suis* strain isolated from the lungs of a diseased pig in Italy [42, 43] was the absence of the 15 K fragment carrying *ermb*, *aph3*-*iiia*, *ant6ia, tet*(40), and *tet*(O/W/32/O) (Figure 2B).

PhageΦSsu1135/10, found in the two Brazilian strains 1135/10 and 1136/10, was integrated downstream of the *rum* gene and upstream of the *glf* gene, and closely resembles the previously described ΦSsUD.1 of a *S. suis* serotype 2 strain isolated from an Italian patient with meningitis [43, 44]. UnlikeΦSsUD.1 bearing *tet*(W) in tandem with *aph3*-*iiia*, *ant6ia*, and *ermb*, ΦSsu1135/10 only carried *aph3*-*iiia*, *ant6ia*, and *ermb*. It is noteworthy that the strains 1135/10 and 1136/10 carried *tet*(O) gene, located outside of ΦSsu1135/10 (Figure 2C).

### Evolution analysis

We assessed the population structure of *S. suis* by analyzing the non-recombinant SNPs in MCG and categorized all strains into 7 MCG groups [15]. In the present study, 4 MCG groups were found among all serotype 9 strains: MCG group 1, MCG group 3, MCG group 4, and 3 lineages of MCG group 7 (MCG group 7-1, 7-2, and 7-3) (Table 1). Taken together, results showed a great diversity of the serotype 9 strain population structure. The two ST16 strains from Brazil and Thailand, shown to be close to the highly virulent strain GZ1 in CGA, were assigned to MCG group 1, which has been previously associated with clinically or epidemiologically important strains [15]. In contrast, all 40 Spanish strains were clustered into MCG group 3, the two remaining Brazilian strains in MCG group 7-3, 10 Canadian strains in MCG group 7-3, eight Canadian strains in MCG group 7-1, two Canadian strains in MCG group 7-2, and one Canadian strain in MCG group 4. Two Canadian strains were non-groupeable. Amongst the four additional serotype 9 genomes, D12 (China), DN13 (China), and S91K (UK) were also in MCG group 7-3, while reference strain 22083 (Denmark) was in MCG group 7-2.

**Figure 3:**
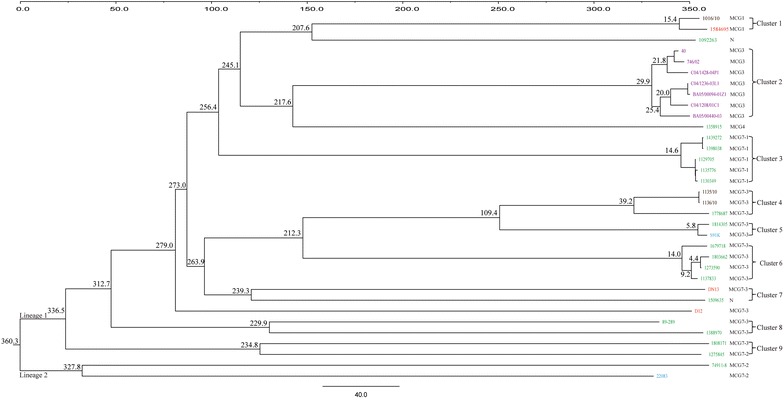
**Phylogenetic relationship and evolutionary time scale of** ***S. suis*** **serotype 9 strains.** The Bayes tree of the isolates was included on top. The inferred emerging years of each clade were marked on the branches. Colors represent geographical distribution of strains: Red: Asia; Brown: Brazil; Green: Canada; Purple: Spain; Blue: European countries (other than Spain).

In present study, we also evaluated the phylogeny of 30 sequenced serotype 9 strains and an additional 4 published serotype 9 genomes using Bayesian evolutionary analysis. The divergence time was estimated using a relaxed molecular clock and the sampling dates of the isolates (range 1986–2015) (Figure 3). We found 1133 genes and 109,815 SNPs in the core genome of the 30 sequenced strains from this study and the additional DN13, D12, S91K, and 22083 genomes already available in public databases. After stripping recombinant SNPs, 48,897 mutational SNPs remained.

Phylogenetic tree using non-recombinant SNPs in core genome revealed two discrete linages (Figure 3). Lineage 1 contained 29 sequenced strains in this study and three additional public genomes, whether DN13 (China), D12 (China), and S91K (UK); meanwhile, lineage 2 was composed of the Canadian strain 74911-8 and the additional public genome of the Danish reference strain 22083. The two distinct lineages diverged 360 years ago, which revealed that evolutionary history of serotype 9 strains may be nearly 400 years old.

Nine different clusters were distinguished in lineage 1. These clusters covered 76.6% (23/30) of the strains as well as the four available genomes and included two to seven strains in each cluster. Cluster 1 contained the two ST16 strains (1016/10 and 1584695). Clusters 2, 3, 6, 8, and 9 had a strong geographical structure signal: Cluster 2 contained all Spanish strains, while clusters 3, 6, 8, and 9 were formed solely by Canadian strains. Cluster 4 included two Brazilian strains and one Canadian strain. Cluster 5 included the public genome of the UK strain S91K and the Canadian strain 1814305. Cluster 7 included the public genome of the Chinese strain DN13n and the Canadian strain 1509635. Generally, the main branches diverged more than 100 years ago, with cluster 1 to cluster 6 emerging in the last 40 years.

### Differences of *cps* locus among strains

**Figure 4:**
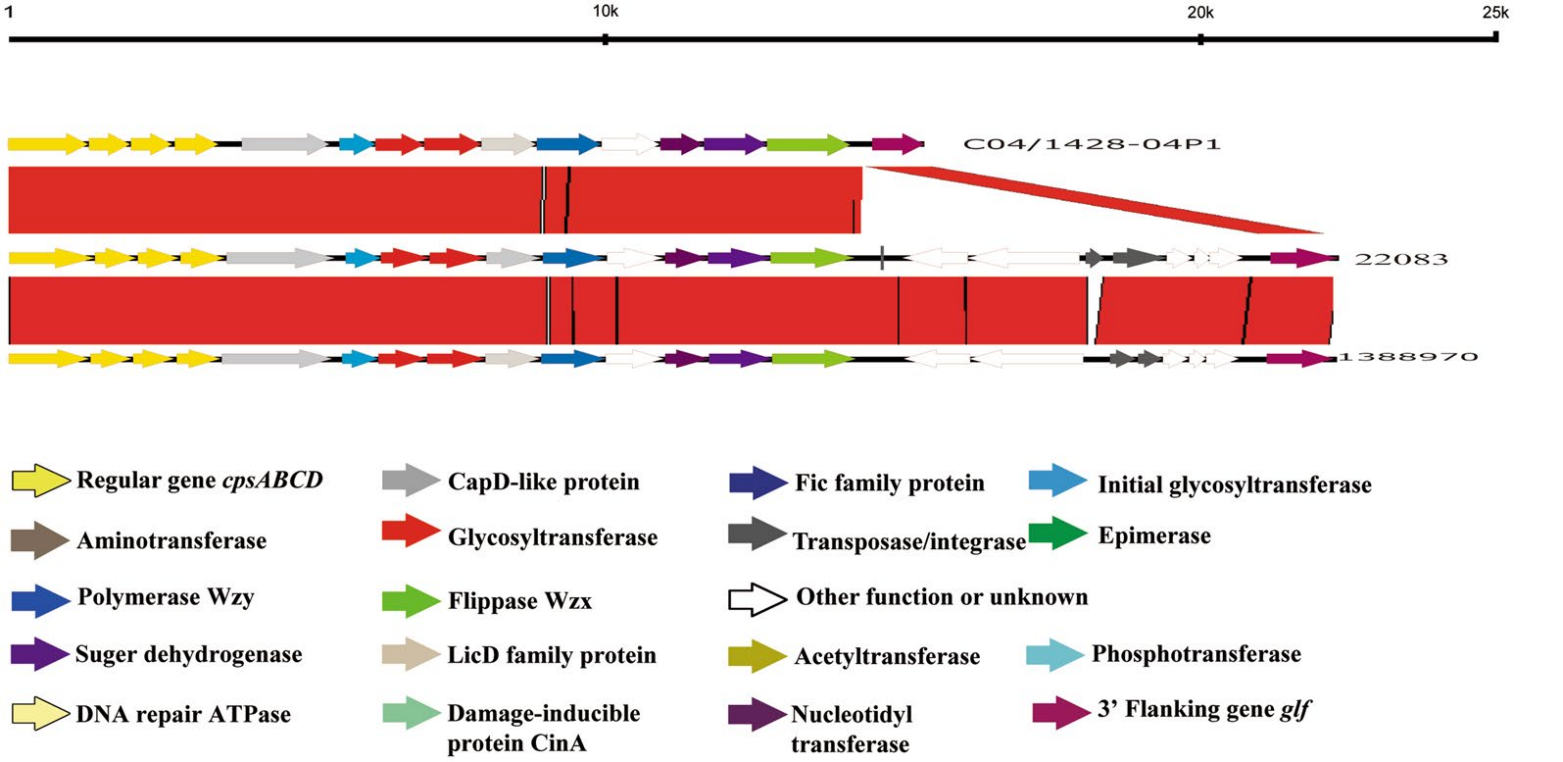
**Comparison of the** ***cps*** **loci among** ***S. suis*** **serotype 9 strains.** Each colored arrow represents a gene whose predicted function is shown in the blown-up panel. The *glf* gene is located on the 3′ side of each locus.

Two types of *cps* loci were found in the 30 sequenced strains from this study (Table 1). Type 1 was prevalent and contained 26 strains. Type 2 only contained 4 strains (89-289, 1275845, 1388970, and 1808171) (Table 1). Compared to the *cps* sequence of the reference serotype 9 strain 22083, Tnp9-1, HG124, HG125, HG126, HG127, and HG128 were absent in the *cps* loci of type 1. *cps* loci of type 2 were almost identical to that of the reference strain 22083, except for a differing repeat region of the transposase gene Tnp9-1 in the 3′ side region (Figure 4). It is noteworthy that the additional serotype 9 published genomes D12, DN13, and S91K also belong to *cps* loci of type 1.

## Discussion

The prevalence of *S. suis* serotype 9 as an etiological agent of porcine disease has increased during the last years in many European countries. However, the variations in pathogenic potential and population structure of serotype 9 strains from different geographical locations have been poorly investigated. Hence, the genomes of a collection of serotype 9 strains from different sources were compared herein. Strains from Spain were chosen since this serotype is the one most frequently isolated from diseased pigs [6]; strains of Canada were included since this serotype is not frequently isolated from diseased pigs [12], whereas those of Brazil were included as controls of strains coming from a completely different environment. Finally, the Thai strain was chosen based on the fact that it is the only strain isolated from a human being so far [13].

Overall, results showed differences between Spanish and Canadian serotype 9 strains. Based on MLST, most Spanish strains included in the present study were either ST123 or ST125 (which belong to the CC61), thus confirming previous reports [37, 45] and clearly showing that these strains are different from those isolated long ago in Spain and in other European countries, which were described to belong to the CC87/CC16 [7, 30, 46]. Indeed, only one Brazilian and the human strain included in this study belong to the ST16 (CC16). In addition, all 40 Spanish strains were clustered into MCG group 3. Conversely, Canadian strains were shown to be highly heterogeneous, with 11 of 13 STs being herein described for the first time. It is important to note that this serotype is not considered highly prevalent in diseased pigs in this country [12]. Interestingly, similar high heterogeneity has been recently reported for serotype 9 strains isolated in Asia, where most strains belong to STs not previously described and also different from those herein observed [22]. Similar to Spanish and Canadian strains analyzed in the present study, no CC16 strain (with a higher zoonotic potential) was detected among Chinese strains [22]. Although the STs of Canadian strains were highly heterogeneous, they mostly belong to MCG group 7. This result may indicate that the reliability of phylogenetic inference based on MLST is adversely affected by the frequency of recombination. Recombination brings in far more base changes to an allele than mutation which adversely affects the inference of true phylogenetic relationships. MCG grouping is an optimal approach to investigate the population structure of *S. suis* based on non-recombinant SNPs in minimum core genome. Results obtained in this study also indicate that Canadian strains undergo high rates of recombination compared to Spanish strains.

In previous studies, CGA proved to be a valuable tool to predict virulence of *S. suis* strains belonging to different serotypes [47]. Indeed, *S. suis* strains were classified as being part of one of the following groups: epidemic or highly virulent (E/HV), virulent (V) or intermediately/weakly virulent (I/WV). Serotype 2 strains GZ1 (ST1) and 89-159 (ST25) were considered as representatives of the E/HV and I/WV groups, respectively [28, 47]. In the present study, only the two ST16 (CC16) strains clustered into group 1 (close to the E/HV group), but in a separate branch from strain GZ1 (CC1). Other common features between the GZ1 genome and ST16 (CC16) strains are the presence of *tet*(W), located in nontransferable genomic island, and the fact that all three were assigned to MCG group 1. Interestingly, the CC16 has been so far considered as being specific to swine [7]. A recent study carried out in the Netherlands also showed high similarity between a zoonotic serotype 2 CC20 group of isolates and porcine CC16 isolates [48]. Taken together, our results also suggest that both ST16 strains possess a certain zoonotic potential that may be lower than that of zoonotic serotype 2 strains. Indeed, a previous study reported that the virulence level of CC16 strains was lower than that of CC1 serotype 2 strains [48]. It is important to note that some genes and RDs which were identified in strain GZ1 were absent from the ST16 strains. Whether or not these genes are responsible for the different virulence potential of these strains remains to be confirmed.

Most serotype 9 strains clustered into group 2 which is phylogenetically close to the I/WV Canadian serotype 2 strain 89-1591. In previous studies, published serotype 9 genomes were phylogenetically distinct from CC1 strains (D12) [16, 48] or clustered into the I/WV group (22083) [47]. Taken together, it may be proposed that the zoonotic potential of serotype 9 strains of groups 2 and 3 is probably lower than that of group 1 strains. Certain genes and RDs reported to correlate with virulence were present in group 1 but absent in group 2 and 3 strains; again, further research is needed to confirm if those genes are responsible for the difference of zoonotic potential of serotype 9 ST16 (CC16) strains. Although CGA analysis indicates that most strains from Spain and Canada may have the same virulence potential for pigs, Spanish strains included in this study are, as mentioned, more homogenous than Canadian strains, which may also be an indication of a higher virulence of the former. However, further studies are needed to evaluate the virulence of representative strains from both groups.

The traditional virulence markers, *mrp*, *epf*, and *sly*, have been mainly associated with serotype 2 strains [19], and to a lesser extent to other serotypes. All strains from Spain were genotyped as *mrp*^NA1^ *epf*^−^*sly*^+^, confirming their homogeneity. Most serotype 9 strains analyzed in the past showed a *mrp*^−^ profile, with a few strains being *mrp*^+^ [37, 49]. In the present study, most strains were *mrp*^+^, represented by the three genotypes already described (EU, NA1, and NA2 [41]). Interestingly, a new genotype was found among Canadian strains, named NA3. Since a role in virulence of the MRP protein has been recently highlighted [50], studies using more isolates from diseased pigs and ill patients will be required to evaluate the real significance of different genotypes of *mrp* and the correlation with the expression of the protein. None of the strains were *epf*^+^, which is in agreement with most previous serotype 9 strain studies [37, 49]. Even if ST16 (CC16) strains are closer to E/H CC1 strains (which usually carry this gene) than other serotype 9 strains, this factor seems to be a clear difference between the two groups. Finally, the *sly* gene was predominately present in all Spanish strains, confirming a higher relationship amongst them, and the two potentially zoonotic serotype 9 strains, but it was absent in over 70% of Canadian strains. Positive *sly* serotype 9 strains have been previously described [37, 49]. Since the suilysin may play important roles in the pathogenesis of the infection [51], its absence may also indicate a potentially lower virulence of Canadian strains.

In the present study, only two strains were free of antibiotic-resistance genes studied. More than 80% of the strains included in this study carried genes associated to resistance to tetracycline, lincosamides, and macrolides, which is in agreement with previous data using *S. suis* strains from North America and several European countries [43, 52–55]. These antimicrobials are still extensively used for therapy and metaphylaxis in the swine industry in different countries, which may contribute to the emergence and spread of their associated resistance. Among antibiotic resistant genes, *tet*(O) was the most prevalent tetracycline resistance gene observed in this study, also previously detected in other Canadian strains [53]. Interestingly, *tet*(M), frequently present in *S. suis* serotype 2 strains from patients in China and Vietnam [52, 53], was almost absent amongst serotype 9 strains.

ICEs are the major contributor to the evolution of drug resistance in *S. suis*. Two novel ICEs, named ICESsu1439272 and ICESsu1679718, were found in Canadian strains carrying *ermb* and/or *tet* (O) genes. Other ICEs have been previously described in *S. suis* strains (ICESsu_SC84_, ICESsu_BM407_2, and ICESsu_32457_) which carry different drug resistance genes [42, 56]. The latter as well as those described in the present study were integrated immediately downstream of the 50S ribosomal protein L7/L12 gene (*rplL*). Having the same ICE insertion sites may indicate that those ICE can spread within/cross-species, serving as a vehicle that enables dissemination of these resistance genes. The rapid increase of resistance genes in strains from pigs may become a severe public health challenge in the near future.

The population structure of *S. suis* serotype 9 strains was composed of two distinct lineages with a common ancestor. They evolved in parallel with lineage 1 becoming dominant. The fact that the serotype 9 reference strain 22083 was grouped into the non-predominant lineage 2 (composed of two strains from different continents) indicated that it does not represent the serotype 9 population, as previously suggested [21]. Moreover, strains in clusters 5 and 6 of lineage 1 were all isolated in more than one country from different continents. It seems that these clusters diverged before spreading. Indeed, a similar mode of transmission has been observed in the largest human outbreak of infection by the epidemic *S. suis* ST7 strain in Sichuan, China [57]. Within lineage 1, there was a rapid population expansion of serotype 9 strains within the last 40 years, possible due to the wide-scale introduction of indoor rearing of meat-producing pigs, as previously suggested [14]. The ongoing inter-continental introduction of pig seeds may have contributed to the expansion.

Finally, different organizations of *cps* loci were observed between strains of lineages 1 (*cps* type 1) and 2 (*cps* type 2). These differences can be attributed to the variable presence of HG124, HG125, HG126, HG127, and HG128. The functions of HG124 and HG125 were related to restriction-modification system, while those of HG126, HG127, and HG128 were unknown. Recently, the chemical composition and structure of type 1 CPS was reported [58]; further analysis of the structure of a type 2 *cps* loci may allow to assess the role of HG124, HG125, HG126, HG127, and HG128. The fact that the reference strain harbors a type 2 *cps* locus also confirms that this strain is rather atypical.

In general, Spanish strains were shown to be more homogenous than Canadian strains suggesting a possible higher virulence of the former. Based on MLST, most Spanish strains included in the present study were either ST123 or ST125, whereas a high number of different STs were detected amongst Canadian strains. However, the distribution of putative virulence factors was, in general, similar in both groups of strains; indeed, more studies are needed to confirm their virulence potential. On the other hand, ST16 (CC16) strains (one isolated from a diseased pig and the other from an ill patient) clearly presented a higher zoonotic and virulence potential. In addition, the presence of ICEs may suggest a possible role in the dissemination of certain drug resistances. The existence of serotype 9 strains may be nearly 400 years old, originating from a common ancestor that further evolved into 2 lineages in parallel. The rapid population expansion of the dominant lineage 1 happened within the last 40 years probably due to the rapid development of the porcine industry.

## Additional files

**Additional file 1.** **Presence/absence of GZ1 genes in genomes of 34** ***S. suis*** **serotype 9 strains and intermediately virulent serotype 2 strain 89-1591 identified in CGA.** The presence or absence of a gene was coded as binary data with gene presence as 1 and gene absence as 0.

**Additional file 2.** **Complete list of non-core virulence-associated genes present in** ***S. suis*** **serotype 9 strains tested.** The presence or absence of a gene was coded as binary data with gene presence as “+” and absence as “−”. Complete genome of GZ0565 was used as reference for BFP66_RS01095, BFP66_RS1875, BFP66_RS7730, BFP66_RS8445, BFP66_RS9410 and Ag like protein (BFP66_04530). Complete genome of GZ1 was used as reference for other virulence genes.

## Abbreviations

CPS: capsular polysaccharides
MCG: minimum core genome
MRP: muramidase released protein
SLY: suilysin
EPF: extracellular protein factor
SNP: single-nucleotide polymorphism
ST: sequence type
MLST: multi locus sequence typing
CGA: comparative genomic analysis
ICE: integrative and conjugative elements
