# Correction to: Genomic comparisons of *Streptococcus suis* serotype 9 strains recovered from diseased pigs in Spain and Canada

**DOI:** 10.1186/s13567-019-0680-9

**Published:** 2019-09-16

**Authors:** Han Zheng, Pengcheng Du, Xiaotong Qiu, Anusak Kerdsin, David Roy, Xuemei Bai, Jianguo Xu, Ana I. Vela, Marcelo Gottschalk

**Affiliations:** 1 State Key Laboratory of Infectious Disease Prevention and Control, Collaborative Innovation Center for Diagnosis and Treatment of Infectious Diseases, National Institute for Communicable Disease Control and Prevention, Chinese Center for Disease Control and Prevention, Changping, Beijing, China; 2 Beijing Key Laboratory of Emerging Infectious Diseases, Institute of Infectious Diseases, Beijing Ditan Hospital, Capital Medical University, Beijing, China; 3 Faculty of Public Health, Kasetsart University Chalermphrakiat Sakon Nakhon Province Campus, Bangkok, Sakon Nakhon Thailand; 4 Swine and Poultry Infectious Diseases Research Center, Faculty of Veterinary Medicine, University of Montreal, Montreal, QC Canada; 5 Departamento de Sanidad Animal, Facultad de Veterinaria and Centro de Vigilancia Sanitaria Veterinaria (VISAVET), Universidad Complutense de Madrid, Madrid, Spain

## Correction to: Vet Res 49:1 (2018) 10.1186/s13567-017-0498-2

In the original publication of this article [1], the author name ‘Pengchen Du’ in author list should be ‘Pengcheng Du’.
